# Crop adaptation to climate change as a consequence of long-term breeding

**DOI:** 10.1007/s00122-020-03729-3

**Published:** 2020-11-22

**Authors:** Rod J. Snowdon, Benjamin Wittkop, Tsu-Wei Chen, Andreas Stahl

**Affiliations:** 1 Department of Plant Breeding, IFZ Research Centre for Biosystems, Land Use and Nutrition, Justus Liebig University Giessen, Heinrich-Buff-Ring 26, 35392 Giessen, Germany; 2 Albrecht Daniel Thaer Institute of Agricultural and Horticultural Sciences, Humboldt University Berlin, Lentzeallee 75, 14195 Berlin, Germany; 3 Institute for Resistance Research and Stress Tolerance, Federal Research Centre for Cultivated Plants, Julius Kühn-Institut (JKI), Erwin-Baur-Strasse 27, 06484 Quedlinburg, Germany

**Keywords:** Genetic gain, Abiotic stress, Breeding progress, Yield

## Abstract

Major global crops in high-yielding, temperate cropping regions are facing increasing threats from the impact of climate change, particularly from drought and heat at critical developmental timepoints during the crop lifecycle. Research to address this concern is frequently focused on attempts to identify exotic genetic diversity showing pronounced stress tolerance or avoidance, to elucidate and introgress the responsible genetic factors or to discover underlying genes as a basis for targeted genetic modification. Although such approaches are occasionally successful in imparting a positive effect on performance in specific stress environments, for example through modulation of root depth, major-gene modifications of plant architecture or function tend to be highly context-dependent. In contrast, long-term genetic gain through conventional breeding has incrementally increased yields of modern crops through accumulation of beneficial, small-effect variants which also confer yield stability via stress adaptation. Here we reflect on retrospective breeding progress in major crops and the impact of long-term, conventional breeding on climate adaptation and yield stability under abiotic stress constraints. Looking forward, we outline how new approaches might complement conventional breeding to maintain and accelerate breeding progress, despite the challenges of climate change, as a prerequisite to sustainable future crop productivity.

## Key message

Breeding is a long-term process. Conventional selection procedures consider plant performance in multiple environments over many years and are thus well-suited for adaptation to climate change. However, modern breeding technologies can help to accelerate the incremental accumulation of positive alleles for “invisible” physiological traits underlying climate adaptation.

## Introduction: the complexity of climate response traits

Crop growth and performance are impacted by a complex interplay with a multitude of interacting environmental (E) and management factors (M), with climate variation explaining a considerable proportion of global crop yield variation (Ray et al. 2015). Both E and M interact strongly with the plant genotype (G), so that higher-order G*E*M interactions must be considered in both breeding and agronomy (Cooper et al. 2020). Considering that G*E*M interactions can affect all manner of physiological processes under quantitative genetic control – for example water and nutrient uptake or transport, dry matter production and partitioning, organogenesis, anthesis, senescence or grain maturation – their impact on source–sink efficiency and yield performance in the face of environmental stress factors is highly complex. A better understanding of the genetic and physiological interplay between molecular and developmental processes underlying crop responses to climate change is therefore considered a key to minimising crop adaptive responses that limit yield potential (Fernie et al. 2020). However, it can be extremely difficult to disentangle the complexity of G*E*M interactions in order to identify useful, selectable component traits for breeding.

For this reason, breeding of arable crops during the past century has generally focused on yield performance as the ultimate outcome of all possible G*E*M interactions. In many crops threatened by the impact of climate change, considerable effort has been invested over recent decades in pre-breeding and introgression programmes that focus specifically on targeted identification and implementation of potentially useful variation for climate adaptation traits. The most intensively studied abiotic stress across all crops is drought, reflecting the major threat of drought to global yield performance as a consequence of climate change.

## Climate change and drought: definitions and implications

In a meteorological definition, drought refers to a period without precipitation affecting an entire region. A distinction is made between regions showing temporary aberrations and those with permanent restrictions of rainfall, the latter is referred to as aridity (Wilhite and Glantz 2009). From an agricultural perspective, drought can be described as a state in which the evapo-transpirational demand exceeds the amount of water able to be taken up by the crop. In this sense, drought stress can be defined as the point at which the soil water content acts as a limiting factor for plant transpiration, namely critical soil theta value $\theta cr$ (Gosa et al. 2019).

This precise definition makes it clear that drought stress is not only the result of an extreme weather event (inadequate replenishment of soil water reservoir for months, depending on the water retention capacity of the soil), but can also be due to diurnal fluctuation of environmental factors, for example a combination of heat with high light intensity, causing an imbalance between rates of root water uptake and canopy transpiration. Therefore, the generic term “drought stress” needs to be understood as a collective of potential drought scenarios (Tardieu and Tuberosa 2010). More detailed elucidation of drought definitions can be found in Wilhite (2000) and Tardieu (2011).

In the face of climatic changes, it is expected that total annual precipitation levels might not necessarily change significantly; however, the frequency and distribution of rainfall is expected to change. In case of phenologically adapted crops, this can result in a situation where the timing of water supply can be unfavourably related to the needs of the plant, for example by a shift towards off-season rain events (Bönecke et al. 2020). Furthermore, even a small increase in spring temperatures may lead to earlier and faster growth of crops, causing them to consume more water earlier in earlier developmental stages and potentially run out of water in early summer (Lian et al. 2020). In this review, we use the term “drought stress” in a broad, generic sense to describe the general negative impact of water deficiency, at any critical timepoints during the plant lifecycle, on crop productivity. In a breeding context, however, drought stress must always be considered in regard to the specific vegetation stage and cannot be generalised as a complex of characteristics. A lack of water in early juvenile development affects plant traits in a completely different manner to effects during at later ontogenetic stages, for example at flowering or grain filling. Accordingly, the search for and improvement of traits with a potentially useful impact on “drought stress tolerance” needs to be contextualised by breeders for defined target scenarios.

Nevertheless, limitations to water and nutrient supply exacerbated by various kinds of drought are a major contributing factor to global yield gaps (Mueller et al. 2012). At the time of writing, the PubMed search term *[drought or “climate change” and (genetics or screening or breeding)]* returned more than 17,500 published articles, of which around 20% referred to the four major global arable crops wheat, rice, maize and soybean. Over half of all these articles were published in just the last five years. However, despite this rapidly growing publication activity, which presumably reflects a simultaneous rise in research activity in the face of expanding climatic challenges for crop production, progress in improving drought tolerance has been no more than incremental in most crops. For example, studies describing individual genes or gene networks purportedly conferring drought resistance in model and crop plants are very frequent (for reviews see Martignago et al. 2020; Shinozaki and Yamaguchi-Shinozaki 2006; Todaka et al. 2015); however, few examples exist for the achievement of significant genetic gains for yield under drought conditions by manipulation of major-effect genes, either using conventional breeding or genetic engineering approaches. One reason for this is that the benefit of a physiological mechanism conferred by a gene or a network normally depends on the drought scenario, which varies between years and sites (Tardieu et al. 2018). Although stress survival traits conferred by mutations in major-effect genes can be beneficial in perennial plants, they tend to be of less use for maintaining yield in highly productive annual crops.

## Modulation of plant adaptation by key regulatory gene families

Nevertheless, some genetic factors may be useful for context-dependent manipulation of stress response characters in cropping environments that have highly predictable stress regimes. For example, single genes with significant positive effects have been identified and manipulated for enhancement of salinity tolerance, for example through induced mutations or gene editing in rice (Takagi et al. 2015; Zhang et al. 2019). As a genetically more complex example, favourable stay-green phenotypes with improved drought tolerance can be achieved by selection for a plant architecture which improves the post-anthesis balance between supply and demand of water for crop growth under water-limited conditions (Borrell et al. 2014; Jordan et al. 2012). Indeed, optimisation of the stay-green trait is arguably the most promising approach to combat drought in major cereals like wheat (Christopher et al. 2016) and sorghum (Borrell et al. 2000a, 2000b). Some major genetic determinants underlying important stay-green loci have been identified. Important examples include the PIN-formed protein (PIN) genes and the vernalisation response (VRN) gene family. Members of the PIN gene family regulate cellular auxin distribution in a multitude of plant tissues and developmental processes (Křeček et al. 2009) and have been shown to regulate various architectural traits contributing to stay-green characters and drought response (Zhang et al. 2012), for example tiller number, root, shoot and leaf size. The VRN gene family contains multiple temperature-responsive genes whose regulation and interaction with other response-pathway genes collectively determine flowering responses to temperature and day-length (Trevaskis et al. 2007). In recent years, increasing evidence has emerged that VRN genes also target genes with central roles in freezing responses, spike architecture and hormone metabolism (Deng et al. 2015). They are also involved in developmental responses to heat stress (Dixon et al. 2019) and the modulation of above-ground and below-ground plant architecture (Voss-Fels et al. 2018).

Although variants of individual PIN and VRN gene family members have been associated with traits implicated in environmental adaptation or stress responses in different cereals (e.g. Abdel-Ghani et al. 2019; Kippes et al. 2015, 2018; Royo et al. 2020), they also impart pleiotropic effects (Voss-Fels et al. 2018; Wang et al. 2010) which may impact yield performance depending on the environment. Accumulation, interaction and gradual selection of beneficial allelic combinations with minor modulatory effects on climate adaption are therefore a logical explanation for long-term optimisation of traits like stay-green characters in the course of crop adaptation to stress environments. Incremental accumulation of genetic variants with small positive effects on multiple physiological traits during breeding (e.g. Voss-Fels et al. 2019b) could be easily explained by ongoing selection for performance and yield stability acting on widespread and subtle allelic variation in the functional domains of large regulatory gene families with a known impact on climate adaptation, like PIN and VRN genes. On the other hand, large-effect mutations with drastic effects tend to be fixed in modern breeding pools. Prominent examples are variants which differentiate the vernalisation requirement between winter and spring-sown ecotypes, major genes for photoperiod responsiveness, or root architecture variants that strongly change source–sink relationships and directly impact yield in target environments.

In the longer term, new approaches to improve photosynthetic efficiency by pathway engineering could potentially introduce new genetic variants that may further help promote cumulative productivity increases. For example, South et al. (2019) showed that introduction of a suite of genetic modifications to the glycolate metabolism pathway can significantly reduce the energy cost of photorespiration and considerably raise photosynthetic efficiency and plant biomass, while Kromdijk et al. (2016) achieved similarly positive effects on photosynthetic efficiency and biomass productivity by engineering photoprotective mechanisms to accelerate responses to fluctuations in light. Both of these studies reported increased photosynthetic efficiency and biomass productivity in greenhouse trials and in field-grown tobacco plants, promising a potentially important impact if comparative systems can be transferred into the complex genomes of major grain crops. However, the plants in both these studies were grown at low planting densities typical for tobacco cultivation and supported by irrigation to ensure adequate water supply. For grain crops growing in a dense canopy without irrigation, translation of enhanced biomass productivity and photosynthetic capacity into grain yield depends strongly on optimised coordination of sink (grain) development and a balanced allocation of limited resources to source and sink development. Although positive effects on water use efficiency are to be expected, the impact of genetically engineered photosynthetic enhancement on grain yield under abiotic stress constraints will depend strongly on an ability to simultaneously optimise and coordinate water and nutrient acquisition, the transition between vegetative and generative growth and the allocation of resources to sink and source organs through the crop lifecycle. The genetics of these processes extend far beyond photosynthetic modulation, so that successful photosynthetic engineering alone cannot provide all answers to enhance breeding progress in the face of climate change. Instead, it appears more likely that genetic gain will continue to be achieved in the foreseeable future by further accumulation of genome-wide, small-effect variants.

## Source–sink trade-offs counter the improvement of single yield components

Yield potential is ultimately determined by the pleiotropic, frequently antagonistic relationships among numerous source–sink characters, which together interact with environmental factors and management practices to balance resource expenditure with crop productivity (Lichthardt et al. 2020; Wu et al. 2019). For example, the gene *WHEAT ABERRANT PANICLE ORGANIZATION 1* (*WAPO1*) has a major effect on quantitative inheritance of row number per rachis (Kuzay et al. 2019; Voss-Fels et al. 2019a), while *GRAIN NUMBER INCREASE 1* (*GNI1*) can increase spikelet fertility (Sakuma et al. 2019). In combination, these two variants might be expected to collectively impart an overall increase in sink capacity, leading to increased grain yield. However, we have found that modern bread wheat cultivars carrying putatively beneficial *WAPO1/GNI1* haplotype combinations do not automatically show the expected positive effect on grain yield, suggesting either a negative pleiotropic interaction or a source limitation that negates any potential epistatic benefit of this combination and renders it neutral in terms of yield selection. Indeed, we found that *WAPO1* variants with more numerous rachis nodes do not impact the number of grains per spike, nor the grain yield (Voss-Fels et al. 2019a). This suggests that fertility repression by *GNI1* may actively prevent excessive grain production. For example, high temperature and/or drought stress are well known to impact pollen fertility in wheat and other cereals (De Storme and Geelen 2013; Saini and Aspinall 1981). Limited water availability in critical generative stages inhibits adequate hydration of the ear tip, which can lead to failure of fertilisation or stunted grains. Wheat produces short-lived, recalcitrant pollen grains that do not enter developmental arrest and are normally highly hydrated when dispersed (Pacini and Dolferus 2019), so that desiccation by pre-anthesis heat and drought can severely limit pollen viability and, consequently, grain development. Voss-Fels et al. (2019b) observed deficits in wheat grain number particularly in environments subject to pre-anthesis heat and drought stress, two constraints which are predicted to increase in importance in European winter wheat as a consequence of climate change. The few examples listed here illustrate the complexities of source–sink relationships even for genetically simple characters. Overcoming the impact of such trade-offs and deficits requires much more comprehensive physiological insight into factors limiting source and/or sink efficiency, as a basis to develop strategies to compensate the deficits through breeding, genetics or crop management. Photosynthetic enhancement may provide one avenue for improvement in this regard, but to understand and deal with source–sink trade-offs it is necessary to gain a holistic view of whole-plant performance in the face of abiotic stress constraints throughout the entire crop lifecycle.

## A “silver bullet” to improve drought performance?

A key reason for the absence of “great leaps forward" in yield performance under drought is that crops display a wide range of physiological drought responses that differ strongly (and often antagonistically) depending on the timing, duration and severity of specific drought events. Rarely are these complex responses governed by single genes and thus amenable to simple selection, marker-assisted breeding, transgenesis or gene editing approaches. Physiologically, a strategy in response to drought, for example an increase in abscisic acid (ABA) production, can trigger several feedbacks in eco-physiological networks. Depending on the genetic and environmental context, this can result in positive or negative effects on short or long-term plant growth patterns (Tardieu and Parent 2017). Therefore, drought responses are normally genetically complex and highly context-dependent, tend to show low heritability and exhibit considerable pleiotropic interactions, particularly with life-cycle traits like flowering time or with grain quality traits.

This means that “drought resistance” per se is arguably a poor stand-alone target trait for breeding, unless the resistance can be associated with a strong yield performance under both stressed and non-stressed conditions (see Guan et al. 2010; Raman et al. 2012 for good examples in rice). Consequently, research efforts which focus on assessment of relative performance between drought-stress vs. non-stress conditions (e.g. Yue et al. 2006) tend to over-value apparently “drought-resistant” genotypes that have an equally poor performance under all conditions, while undervaluing “susceptible” genotypes which in fact perform reasonably well under severe drought but poorly in relation to optimal conditions. On the other hand, the ecological concept of “response diversity” (Kahiluoto et al. 2019), describing a strong fluctuation of genotype-by-environment responses as a fundamental basis for selection, is also poorly suited to plant breeding for agricultural production systems (Snowdon et al. 2019) because it overlooks the need for consistent maximisation of performance under all possible conditions.

Drought effects on crop productivity are frequently compounded by heat stress effects, because the two stresses commonly (though not always) occur in tandem, and they can also be further compounded by stress from high light (Zandalinas et al. 2020). However, different stress responses are not always genetically related, so that genetic dissection of one stress response necessitates careful control or monitoring of the other; this requires stable and predictable environments for testing or selection. Due to the high complexity of potential interactions between different quantitative stress response traits, and the considerable cost and effort involved in effective evaluation and selection for drought/heat tolerance in the context of grain yield, the pragmatic approach of breeders to is generally to select for yield performance and yield stability across a broad range of environments which encompass varying degrees of stress for a range of abiotic stress factors.

## Conventional breeding worked well so far

This classical breeding approach was the fundamental basis of the more or less linear increases in genetic gain in the course of the past century that have been reported by a multitude of studies in different regions for many different crops, for example wheat (Crespo-Herrera et al. 2018; Fischer and Edmeades 2010; Peltonen-Sainio et al. 2009; Sanchez-Garcia et al. 2012), maize (Badu-Apraku et al. 2015; Ci et al. 2011; Duvick 2005; Russell 1991), rapeseed (Stahl et al. 2017, 2019), soybean (Rincker et al. 2014; Ustun et al. 2001), barley (Laidig et al. 2017), sugar beet (Loel et al. 2014) and rye (Laidig et al. 2017). Breeding durations for arable crops, from the initial cross until the subsequent fixation of desirable genetic components from the parents into a stable cultivar, can frequently span up to a decade. Hence, conventional selection processes that test breeding progenies in multi-environment phenotypic evaluations over many years appear intrinsically well-suited to select for adaptation to gradual climatic changes, which also develop over the course of several years or a few decades. On the other hand, should severe yield repression imparted by severe drought and heat (Lobell et al. 2011) become the norm rather than the exception in important temperate cropping regions, breeding progress for these specific target traits must necessarily accelerate at a greater rate than previously to compensate serious productivity losses in the face of climate change. Therefore, to optimise future genetic gain in the face of climate change, it is necessary to consider how selection processes might be further optimised to more effectively capture genome-wide, small-effect variance that positively affects long-term adaptation to key abiotic stressors, without inadvertent introduction of negative pleiotropic effects on yield performance.

Since genetic diversity provides the essential basis for effective maintenance of long-term genetic gain, breeding programmes must take care to manage selection intensity and effective populations sizes in order to reduce the risk of losing potentially useful adaptation alleles for future climatic scenarios. Modern hexaploid wheat breeding pools exhibit considerable founder effects, visible as differential subgenomic diversity patterns associated with directional selection for important phenological, plant height or resistance traits (Hao et al. 2020; Voss-Fels et al. 2015; Zhao et al. 2019). For example, Voss-Fels et al. (2016) found that linkage drag, caused by preferential selection acting on a locus impacting post-vernalisation anthesis in European winter wheat, had eroded diversity for two closely linked QTL for root biomass, strongly restricting phenotypic diversity for root traits that could be potentially important in future cultivars in the face of climate change. Recycling allelic diversity from non-adapted primary-gene pool sources or exotic wild relatives (He et al. 2019) is therefore still a vital aspect of modern breeding programmes for long-term maintenance of genetic diversity.

## Has breeding progress for yield diminished genetic diversity for climate adaptation in crop gene pools?

A frequent assumption in relation to genetic diversity in modern, elite crop gene pools is that the focus of breeding on high productivity might inadvertently lead to loss of important genetic diversity for climatic adaption traits which might be potentially essential to ensure sustainable breeding progress in the face of climate change. Underlying this assumption is a legitimate concern that the consequences of climate change are a relatively new phenomenon in the world’s most productive arable cropping areas, where the relatively predictable and favourable climatic conditions that dominated the past century were a key factor in the establishment of extremely productive agricultural production systems. Today, however, extended periods of serious abiotic stress during key periods of crop growing seasons are also threatening regions that have benefitted from a century of enormous breeding progress under favourable climate conditions.

Plant breeding programmes targeting high-yield cropping systems logically focus on maximising yield under the predominant production conditions. Because water and nitrogen supply are major limiting factors for grain yields, historical yield increases are often associated with greater water and nitrogen consumption rather than with genetic gain (Sinclair and Rufty 2012). This is sometimes interpreted to suggest that modern elite cultivars have reduced water and nutrient efficiency, even though empirical studies show the opposite (e.g. Badu-Apraku et al. 2015; Hatzig et al. 2015; Voss-Fels et al. 2019b). Since high-yielding conventional cropping systems generally optimise plant nutrition and health using adequate applications of mineral fertiliser and chemical plant protection, it might be expected that genetic variants associated with performance under sub-optimal input conditions might be eliminated from modern breeding pools by genetic drift due because they confer no selective advantage. For example, Kahiluoto et al. (2019) asserted that a reduction in what they interpreted as “response diversity” in European winter wheat cultivars was caused by a reduction in genetic diversity through breeding, although this claim was not supported with data. In direct contrast, however, various empirical studies have in fact shown that genetic diversity in elite European wheat gene pools has actually not declined in recent decades (van de Wouw et al. 2010; Voss-Fels et al. 2019b; Würschum et al. 2018).

Despite evidence suggesting otherwise, the focus of breeding on improvement of grain or biomass yield is frequently interpreted by non-breeders to be associated with a neglect of traits related to sustainability (for example disease resistance, nutrient or water use efficiency), likely due to a misconception that these traits are not relevant in high-yielding conditions because yield-suppressing factors are able to be minimised by intensive management. This chain of argumentation is often expressed in popular media sources, but rarely supported by empirical data in peer-reviewed publications. In stark contrast to this hypothesis, however, studies that empirically examined retrospective breeding progress in different crops have actually concluded that the opposite situation appears to be the case (Badu-Apraku et al. 2015; e.g. Chen et al. 2013; Voss-Fels et al. 2019b). Some studies also simultaneously shed interesting light on the genetic architecture of long-term yield progress for crop production under sub-optimal growth conditions, providing insights which may help guide future breeding to maintain breeding progress in the face of climate change. Such examples demonstrate the importance of empirical data to document the consequences and potential of breeding for improved adaptation to sub-optimal production environments.

## What can we learn from retrospective breeding progress?

In a major recent example in which retrospective breeding progress was investigated in winter wheat from Western Europe, an intensive cropping region where wheat grain yields are among the highest in the world, Voss-Fels et al. (2019b) analysed a panel of around 200 elite accessions released during the past five decades, selected to represent the most successful and highest performing cultivars during their respective periods of registration. The cultivars were grown in a large-scale experiment across six locations for multiple years, in order to analyse genetic gain for grain yield and compare performance across a multitude of component traits, including yield and biomass components, grain quality, resistance and physiological traits. Uniquely, this study repeated the same experiments side by side under contrasting managing conditions, including reduced nitrogen fertilisation and fungicide-free treatments. These reduced intensity treatments enabled a highly detailed assessment of how breeding for high yield in the optimised, high-input agricultural production of Western Europe has impacted the ability of modern cultivars to respond to adverse conditions with sub-optimal plant protection and/or nitrogen nutrition.

**Fig. 1:**
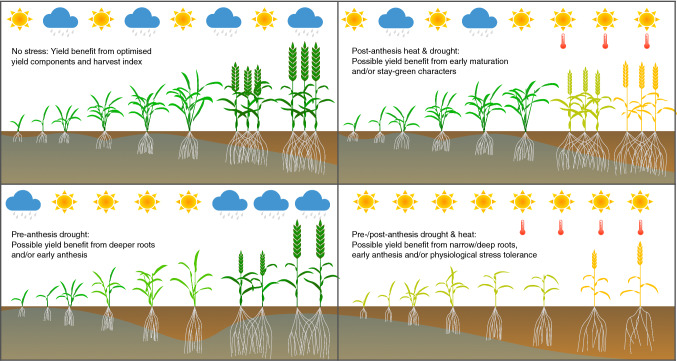
Climate change can potentially have very different impacts on crop performance depending on the time-point and duration of drought events and whether drought stress occurs in combination with heat stress or alone. Depending on the type and intensity of stress, different genetically determined responses may be more beneficial for the performance of a cultivar. This means there is no single, simple per se solution for breeders to overcome the potential impacts of drought stress caused by climate change. Selection for performance and yield stability under the expected stress scenarios that occur commonly in a particular target region may be the best way to maintain crop performance while mitigating risks. Yield selection in target environments also improves complex stress response traits and yield stability (Voss-Fels et al. 2019b)

In accordance with other studies of retrospective breeding progress mentioned above, the results of Voss-Fels et al. (2019b) showed a clear pattern of linear genetic gain for yield performance of cultivars over time as a result of breeding. Furthermore, the incremental gains in yield over time were also reflected in corresponding patterns of incremental improvement in almost all other traits reported (Fig. 1a). Most interestingly, these included not only traits for which ongoing selection in long-term breeding would be expected to result in continuous improvement, for example important fungal disease resistances, grain quality, harvest index and key primary yield components like grain number per spike (Fig. 1b). Moreover, clear evidence for ongoing, incremental genetic gain could also be observed in complex, low-heritability traits for which breeders do not have the resources to devote to dedicated long-term selection. For example, nitrogen use efficiency and photosynthetic efficiency was found to be superior in newer cultivars than their older counterparts, although these traits are both very difficult and expensive to phenotype in multi-environment trials of large breeding populations. The obvious explanation is that traits impacting a more efficient utilisation of resources (e.g. water, nutrient and light) are important contributors to overall performance in the high-competition environments of intensive cropping systems. Conversely, it might be expected that elucidation and targeted consideration of relevant physiological component traits and their underlying quantitative genetic architecture in breeding activities could help accelerate genetic gain for yield stability.

Presently, however, a routine assessment of complex physiological or developmental traits associated with climate adaptation is beyond the scope of most breeding programmes. Instead, breeders rely on selection for yield as a simple-to-measure end-point phenotype which directly reflects the economic value of a selected genotype. With suitably replicated field evaluations in multiple locations, breeders gain accurate estimations of genetic potential for yield and yield stability that enable reliable, direct selection for their most important target trait. In contrast, it is very challenging for breeders to effectively identify and exploit novel genetic diversity for adaptation of crops to highly specific stress situations, for example post-anthesis drought, without potentially compromising yield performance in environments where the stress factors are absent or different. Selection for combinations of beneficial, context-dependent component traits requires detailed knowledge about how key yield-determining or yield-limiting processes are impacted by G*E*M interactions. These can provide a modelling framework to identify climatic factors impacting yield gaps and coordinate breeding and crop management in order to lessen the impact of key abiotic stress factors (Cooper et al. 2020).

## Dissecting the basis of retrospective breeding progress to target future climate adaptation

Individual component traits (e.g. root, leaf, spike and canopy traits or stay-green characters) and specific physiological processes contributing to grain yield (e.g. water/nutrient uptake or translocation, biomass/grain development and photosynthesis) are frequently studied in considerable detail in selected plant genotypes. To adequately understand genotype–environment interactions with abiotic stress and their impact on reproductive capacity and grain yield, it becomes necessary to elucidate “hidden” traits and pleiotropic interactions by detailed temporal phenotyping across critical crop growth periods. However, detailed investigations of dynamic temporal phenotypes in representative crop populations or breeding progenies are much more challenging than single time-point phenotyping of simple target traits. Although analyses of dynamic phenotypes in large breeding populations are very rare to date, new advances in digital phenotyping and drone-based remote sensing are beginning to deliver a level of detail that can help uncover hidden genetic variants impacting the complex genetics of yield and stress responses (for examples, see Chen et al. 2014; Chenu et al. 2018; Knoch et al. 2020; Stahl et al. 2020; Tardieu et al. 2017). Integrating recent high-throughput phenotyping, modelling approach and automatic computational data-processing pipeline have shown promising potential to obtain heritable physiological traits related to productivity for future breeding programmes (Chen et al. 2019).

Extensive retrospective datasets documenting breeding progress, like those described in Fig. 1, are a valuable resource to identify genome-wide haplotype diversity for relevant source–sink traits involved in genetic gain for resource-efficient crop productivity. For example, yield gains in modern cultivars associate with an improved source efficiency, due to component traits like high water and nitrogen uptake efficiency, high biomass potential, high radiation interception/radiation use efficiency or a remarkable sink strength imparted by a high number of grains per unit area (Voss-Fels et al. 2019b). However, cultivars carrying such putative physiological advantages do not always exhibit the expected positive impact on grain yield: Instead, such genotypes often show sink deficits in terms of irregular grain formation, spatio-temporal variability for grain development or other source–sink trade-offs (Lichthardt et al. 2020).

Long-term yield gains through breeding progress typically correspond with incremental, genome-wide elimination of alleles with small, detrimental effects as a response to selection (Voss-Fels et al. 2019b; Yang et al. 2017), and this phenomenon also has a positive impact on breeding progress in abiotic stress environments (e.g. Abdulmalik et al. 2017). The small effect sizes inferred by this kind of positive, ongoing selection reflect Fisher's infinitesimal model of evolutionary adaptation (Fisher 1930), which presumes that complex traits are under control of many thousands of quantitative trait loci (QTL) that have only small individual effects. On the other hand, QTL with larger effects, which contradict the infinitesimal model in breeding populations (Mackay 2001), are generally rapidly fixed for the most beneficial alleles in elite, adapted cultivars (e.g. Voss-Fels et al. 2019b). This observation corresponds to Kimura's modifications of Fisher's adaptation model (Kimura 1979, 1991), which describe how favourable large-effect mutations have a high probability of fixation. However, as noted by Orr (1998), remaining variants and new mutations tend to have only very small effects when an organism is close to its optimal state, as is the case in high-performing elite cultivars resulting from human selection during breeding. Maintenance of breeding progress in the face of climate change will rely heavily on an ability to reveal and combine genome-wide, small-effect loci controlling traits with a cumulative beneficial impact on resource efficiency and yield performance.

Recently, Mahmoud et al. (2020) presented a novel quantitative genetic approach that uses retrospective datasets during the course of breeding progress to identify traits that have been under selection at large numbers of loci. The method relates additive effects of genome-wide SNP markers to allele-frequency changes over time, using a composite statistic (Ghat) that can identify significant evidence of selection on a given trait, even in relatively small panels of cultivars or breeding lines. Such methods open the possibility for large-scale retrospective analysis of digital, physiological, morphological, regulatory and/or metabolic traits in carefully preselected genotype panels to pinpoint trait complexes that have been subject to particularly strong indirect selection in the course of long-term breeding progress for grain yield. Such knowledge could provide a theoretical framework to test new selection regimes based on stress response and yield stability characters which were previously invisible to plant breeders, but could hold the key to accelerating genetic gain in the face of increasing environmental challenges. Genomic selection strategies based on relevant trait complexes which were not yet accessible for breeders can expand the repertoire of breeding tools to combat the impact of climate change.

## Conclusions

“Silver bullets” to breed for climate change, in the form of novel or induced genetic variants for beneficial phenological and physiological responses, can certainly play an important role in context-dependent crop stress adaptation, and new tools like gene editing provide breeders with completely new opportunities to accelerate the implementation and stacking of beneficial variants for important target traits. However, the majority of major-effect genes for key adaptation traits are generally rapidly fixed in high-performing modern crop gene pools, and pleiotropic interactions associated with severe changes in plant phenology mean that breeders need to be cautious when promising huge returns from technological breakthroughs on a single-gene level. On the other hand, conventional selection processes in plant breeding, driven by testing and selection in diverse environments over many years, have proven well-suited for gradual adaptation of modern cultivars to changing environmental conditions—just as the same approach was enormously successful in adapting globally successful crops to diverse eco-geographical and climatic conditions that go far beyond the natural habitat range in their centres of origin.

Incremental accumulation of beneficial alleles for complex quantitative traits impacting yield and quality has been the fundamental basis of historical breeding progress in all crops, and this process can be expected to also play a major role in crop adaptation to climate change in future. However, retrospective breeding progress for climate adaptation was in most cases simply the positive by-product of long-term, multi-environment selection for high yield and cultivar registration policies that encourage high yield stability (Snowdon et al. 2019). This is because breeders in the past did not have access to the resources and tools that today enable effective elucidation, genetic dissection and targeted selection of key adaptive target traits with small, genome-wide effects under complex genetic control. Opportunities to capture and exploit the temporal complexities of important environmental lifecycle traits are today considerably more accessible. Automated phenotyping, remote sensing and image analysis are improving continually, while genomic selection strategies are expected to grow in power with an expanding phenomic and genomic data basis that makes it increasingly feasible to exploit the vast power of new prediction methods based on artificial intelligence and “deep learning”. As the world population continues to expand and demand for crop products continues to rise, yield performance as the outcome of G*E*M interactions will certainly continue to be the most valuable target trait for breeding progress in the face of climate change. Simultaneously, the methods with which breeders maintain yield progress are today being increasingly enriched by powerful new techniques and technologies that facilitate identification, introgression and recombination of novel, genome-wide diversity for complex adaptive traits in a high-yield context. Even as this repertoire of breeding technologies expands, however, classical breeding theory and methodology will continue to secure long-term breeding progress to ensure ongoing crop productivity in the face of climate change.
